# An Active Learning Method Based on Variational Autoencoder and DBSCAN Clustering

**DOI:** 10.1155/2021/9952596

**Published:** 2021-07-30

**Authors:** Fang Chen, Tao Zhang, Ruilin Liu

**Affiliations:** School of Information and Mathematics, Yangtze University, Jingzhou 434023, China

## Abstract

Active learning is aimed to sample the most informative data from the unlabeled pool, and diverse clustering methods have been applied to it. However, the distance-based clustering methods usually cannot perform well in high dimensions and even begin to fail. In this paper, we propose a new active learning method combined with variational autoencoder (VAE) and density-based spatial clustering of applications with noise (DBSCAN). It overcomes the difficulty of distance representation in high dimensions and prevents the distance concentration phenomenon from occurring in the computational learning literature with respect to high-dimensional p-norms. Finally, we compare our method with four common active learning methods and two other clustering algorithms combined with VAE on three datasets. The results demonstrate that our approach achieves competitive performance, and it is a new batch mode active learning algorithm designed for neural networks with a relatively small query batch size.

## 1. Introduction

In practical issues, the amount of labeled data is relatively small and the vast majority is unlabeled. It is often unrealistic to consume a lot of human resources and expensive cost to annotate unlabeled data for the annotation budget is limited. Active learning [1] is born out of this problem, which is one field trying to address the difficulties in data labeling. It assumes that the collection of data is relatively easy, but the labeling process is costly. It tackles the problem of which samples we should label to result in the highest improvement in test accuracy under a fixed labeling budget. Existing active learning algorithms are mainly divided into two categories, query-synthesizing [2–4] and query-acquiring [5]. Query-synthesizing approaches use generative models to generate informative samples whereas query-acquiring algorithms use different sampling strategies to determine how to select the most informative samples.

In this paper, we mainly pay attention to query-acquiring methods. One of the query-acquiring algorithms is uncertainty-based method [6, 7]. Settles [8] mentioned that the classifier gives every unlabeled data a probability score which represents the uncertainty belonging to its class, and then we choose the data with the highest uncertainty. Lewis and Gale [9] argued that uncertainty-based methods perform well on a large and diverse set of datasets. Yarin et al. [10] also proposed a Bayesian active learning framework, in which Bayesian neural networks [11] are used to estimate uncertainty. Later, Gissin and Shai proposed a discriminative active learning method [12] in which an uncertainty idea is also used to sample the unlabeled data with the top-K highest possibility when the batch size is relatively large. The other type of query-acquiring methods is representation-based approach, which relies on selecting few examples by increasing diversity in a given batch. Ozan and Savarese [13] and Suyog and Grauman [14] adopted this idea in their experiments. However, it seems to be ineffective to use distance-based representation methods like Core Set for high-dimensional data because the distance concentration phenomenon appears in the computational learning literature with respect to p-norms in high dimensions [15].

To address the difficulty of high-dimensional distance representation, Sinha proposed a variational adversarial method [5] by learning a latent space using a VAE [16, 17]. VAE is called the perfect representation learning method of high-dimensional data, which has been proved effective. Therefore, we adopt VAE in our approach to solve the difficulty of distance representation especially for digital images [18]. VAE plays a great role in our method, which perfectly represents high-dimensional data into the low-dimensional representation in latent space, preventing the occurrence of the phenomenon of*p*-norm distance concentration of high-dimensional data. And in common clustering approaches [19–22], DBSCAN [23] is good at identifying the noise and discovering arbitrarily shaped clusters without knowing the number of cluster classes to be formed in advance. Therefore, we propose a new active learning strategy combined with VAE and DBSCAN clustering for considering their advantages. What is more, excellent active learning models are usually based on large query batch sizes. But they may ignore the performance of a relatively small query batch size based on small sample problems. So we conduct our experiment on a relatively small query batch size to see the performance.

The rest of this paper is organized as follows.  Section 2 illustrates the problem setting and the brief definition of related methods.  Then Section 3 introduces our proposed model and the algorithms. Subsequently, the experimental results based on a relatively small query batch size are provided in Section 4. In the end, we draw a conclusion in Section 5.

## 2. Related Notion

### 2.1. Problem Definition

We define the problem of active learning formally as follows. Given a labeled pool (*X*_*L*_, *Y*_*L*_) and a much larger unlabeled pool *X*_*U*_, we are aimed to sample the most informative unlabeled data *x*_*U*_ from *X*_*U*_ by iteratively querying a fixed sampling budget in order to train the most label-efficient model in an active learning task. During this progress, *n* number of unlabeled data will be sampled by an acquisition function and be annotated by the oracle. This is an iterative process which is done until a certain stopping criterion is reached, such as the desired amount of samples or test accuracy.

### 2.2. Related Methods

With respect to VAE, the parameters are trained by two loss functions if *N* training samples are given. One is the reconstruction loss *L*_1_, which forces the reconstructed sample $\overset{\wedge }{x_{i}}$ to match the original input sample *x*_*i*_. Here, we use the cross entropy for measurement:

(1) $$\begin{array}{c} L_{1}=-\sum_{i=1}^{N}\left[x_{i}\cdot \;\log\overset{\wedge }{x_{i}}+\left(1-x_{i}\right)\cdot \;\log\left(1-\overset{\wedge }{x_{i}}\right)\right],\;\text{where }i=1,2,\ldots ,N. \end{array}$$

In addition, we are aimed to make $\overset{\wedge }{x_{i}}$ as close as possible to *x*_*i*_ itself. That is to say, it can retrieve as much of the original information as possible through decoding. The other one is regularization loss *L*_2_, which helps to learn latent space with good structure and reduces overfitting on the training data. The formula is shown as follows:

(2) $$\begin{array}{c} L_{2}=\frac{1}{N}\sum_{i=1}^{N}\left[1+\log\left(\sigma_{i}^{2}\right)-\mu_{i}{}^{2}-\exp\left(\log\left(\sigma_{i}^{2}\right)\right)\right],\;\text{where }i=1,2,\ldots ,N. \end{array}$$

In the formula above, the mean *μ*_*i*_ and the variance *σ*_*i*_^2^ of every input data are computed by an encoder. Then the encoder learns a low-dimensional latent space for the underlying distribution *N*(*μ*_*i*_, *σ*_*i*_^2^) using a Gaussian prior. Therefore, we obtain the total objective function for VAE as follows:

(3) $$\begin{array}{c} L_{\text{VAE}}=L_{1}+L_{2}. \end{array}$$

Then, we minimize the total loss *L*_VAE_ and a high efficient variational autoencoder model will be obtained after rounds of optimization. It is worth mentioning that the reparameterization trick is adopted by sampling a data *ε* from a standard normal distribution *N*(0,1). Then, we could sample the low-dimensional data *z*_*i*_ randomly in the latent space:

(4) $$\begin{array}{c} z_{i}=\mu_{i}+\sigma_{i}⊙\varepsilon , \end{array}$$

in which *ε* is a very small random tensor and ⊙ is the element-wise product.

**Figure 1:**
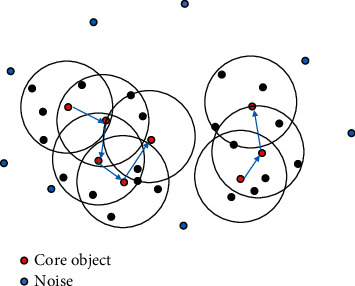
DBSCAN clustering if MinPts = 5.

In our approach, clusters in DBSCAN are defined as the largest set of points connected by density. A region with sufficient density can be divided into a cluster, and points that are not clustered are called noise. The basic process of DBSCAN is as shown in Figure 1.

## 3. Proposed Model

### 3.1. The Combination of VAE and DBSCAN Clustering

**Figure 2:**
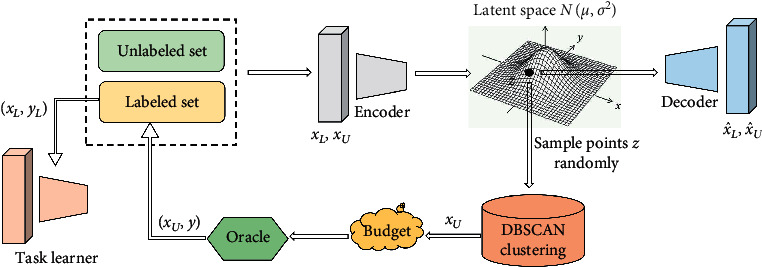
An active learning method based on VAE and DBSCAN clustering.

In this paper, we suggested a new active learning method based on VAE and DBSCAN clustering on a relatively small query batch size. We motivate our method with a simple idea. First, VAE learns a valid low-dimensional latent feature space for the underlying distribution using a Gaussian prior from the labeled and unlabeled pool, whose space is a mixture of the latent features. Then we adopt density-reachable clustering DBSCAN to remove noise in the initial clusters, and we sample the most valuable unlabeled data in high density also in different clusters from the latent space. The framework of our model is shown in Figure 2.

In detail, the VAE model in our experiment is combined by a convolutional neural network and a deconvolutional neural network. The convolutional one named encoder consists of four convolutional layers, one flatten layer, and three fully connected layers in order. And the deconvolutional one named decoder is relatively simple, consisting of a fully connected layer and two convolutional layers. We update VAE by descending stochastic gradients. After iteration and updating, we obtain the trained parameters *θ*_VAE_ also the trained model VAE. In the end, VAE learned a two-dimensional latent feature space efficiently.

The low-dimensional feature vectors learned by VAE serve as the input data for the following clustering methods. In DBSCAN, we start sampling unlabeled points in the clustered classes after an initial unlabeled core object is computed. Here, we note that we remove the noise at the first step. Suppose the needed amount of unlabeled data is *C*, the total number of all unlabeled density-reachable points in all clusters by DBSCAN is required to be as close to *C* as possible. The purpose is to ensure that different types of high-density unlabeled data can be retrieved as much as possible. After that, *C* number of corresponding original unlabeled data will be sampled by an acquisition function and to be annotated by Oracle. Then put them into the original labeled pool and use the task learner to test the mean accuracy of our proposed model.

### 3.2. Algorithms

For clarity, we describe our method in Algorithms 1 and 2.

## 4. Experiments

### 4.1. Dataset and Task Module

We evaluated our method on three typical image datasets MNIST [24, 25], Fashion-MNIST, and CIFAR-10. The first one MNIST contains 60,000/10,000 (train/test) handwritten digit images (0∼9) with the resolution of 28 × 28. And Fashion-MNIST is an alternative version of MNIST which has the same size as MNIST with frontal images of different items from 10 categories such as shirts, pants, sandals, and bags. They are also the single channel images with the same size of 28 × 28. CIFAR-10 contains 10 categories of images such as airplanes, automobiles, birds, and cats. Different from the two datasets above, images in CIFAR-10 are 3-channel color RGB images with the size of 32×32. Not only the noise is very large, but also the proportion and characteristics of the object are not the same, which brings great difficulties for recognition.

We used the classic LeNet architecture [26] as our task module for MNIST and Fashion-MNIST, while for CIFAR-10 task we used VGG-16 (Simonyan and Zisserman, 2014) as our architecture. And we took a simple convolutional network just similar to LeNet architecture as the second task module when comparing the different clustering active learning algorithms. This module consists of three convolutional layers, two maximum pooling layers, a flatten layer, and two fully connected layers in order.

### 4.2. Baseline Algorithms

To demonstrate the effectiveness of our method, we took the following four common active learning methods and also the random sampling as the baseline algorithms. They are briefly described as follows:

- Random: the query batch is chosen uniformly at random
- Uncertainty: uncertainty sampling with minimal top confidence
- Core Set: in every round of query, sample the unlabeled data which are of the largest distance from the labeled set and then add to the labeled pool
- EGL: estimated gradient length
- Bayesian: Bayesian uncertainty sampling with minimal top confidence

Furthermore, in order to comparing the performance of different clustering algorithms [27] in active learning task, we replaced DBSCAN by two classic and widely used clustering algorithms: K-means [28] and Mean shift [29]. And they are also compared with random sampling. These clustering methods in our experiment were based on a two-dimensional feature dataset learned by VAE which are extracted from the three datasets listed above. Their brief description is as follows:

- K-means with VAE: after VAE, we sample the two-dimensional data around the cluster centers according to K-means clustering rule and a given radius.
- Mean shift with VAE: after VAE, solve for a vector that moves the center of the circle in the direction with the highest density of the dataset, and the average position of the points inside the circle is found as the new center position in each iteration. We choose the points around these centers.
- DBSCAN with VAE: our method, detailed above.

### 4.3. Implementation Details

**Figure 3:**
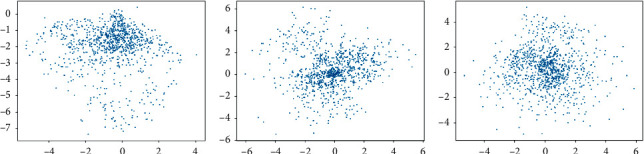
The two-dimensional feature points in latent space in different epochs: (a) 20 epochs, (b) 200 epochs, and (c) 2000 epochs.

**Figure 4:**
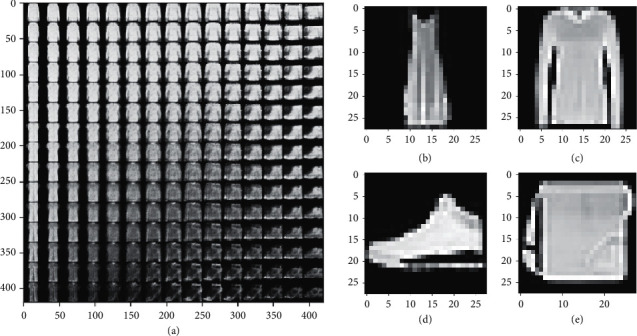
Some original images corresponding to the data points in latent space.

In our VAE model, the learning rate was selected by default as *α*=1 × 10^−3^. What is more, we chose Sigmoid as our optimizer. Based on a relative small query batch size, it shows that when epochs reach about 400, the learning loss almost does not decrease and the distribution of latent feature space learned by VAE comes to be stable. In other words, we set the number of training epochs to 400 to prevent overfitting.  Figure 3 shows the two-dimensional feature points learned by VAE in different epochs. After VAE is trained, a certain number of low-dimensional data samples were extracted by its well-trained encoder. We checked some corresponding original images as shown in Figure 4 (taking Fashion-MNIST as an example).

In order to observe the performance of active learning algorithms on a relatively small query batch size, we tried to sample 100 low-dimension data points randomly from the latent space learned by encoder in VAE as our clustering dataset for DBSCAN, including 10 labeled data and 90 unlabeled data, and our query batch size is set to 5 which is relatively small. Finally, we obtained the needed amount of unlabeled density-reachable points in different clusters after removing the noise. It is particularly noted that we adjust the parameters to make the total number of unlabeled points in all density-reachable clusters to be as close as possible to the needed amount of data for annotations.

### 4.4. Results

**Figure 5:**
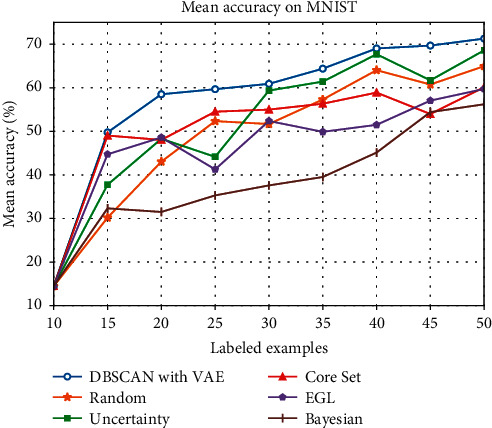
Mean accuracy (%) of different active learning methods on MNIST.

In our experiment, we used the test accuracy as our metric to evaluate the performance. The results are averaged from 20 runs in order to ensure statistical validity. By the results in Table 1, we plot the test accuracy of experiments using four different active learning methods on MNIST in Figure 5. The results demonstrate that, in MNIST, our method, DBSCAN with VAE, performs better on small query batch size than the active learning methods listed above. Figure 5 also shows that some methods perform on par or worse than random sampling. This may be the reason that the sample size is relatively small and the randomness is strong, which reduces the gap between each sampling method and random sampling. It may also be because some methods are more suitable for large query batch sizes.

We also notice that, except for EGL performing well than random sampling on very small batch sizes below 20, most algorithms listed above including random sampling consistently outperform EGL. As for EGL, a possible explanation for the discrepancy between these results may be the architecture used for the different tasks because EGL uses the gradient relative to the model parameters as the score function [12]. Among all the methods above, Bayesian performs the poorest and it may be because Bayesian is more suitable for large training datasets.

**Figure 6:**
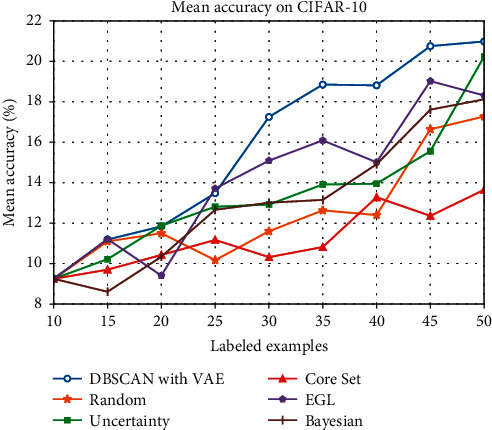
Mean accuracy (%) of different active learning methods on CIFAR-10.

Next, we did a comparison test on CIFAR-10. The result is in Table 2 and we plot it in Figure 6.

As can be seen from Figure 6 on CIFAR-10, Core Set often performs worse than random sampling, and it is also possibly because the random advantage is greater than Core Set when the sample size is small. And when the size is below 25, our approach shows little advantage than other methods, but our approach leads all the way obviously when the number of labeled samples is over 25.

**Figure 7:**
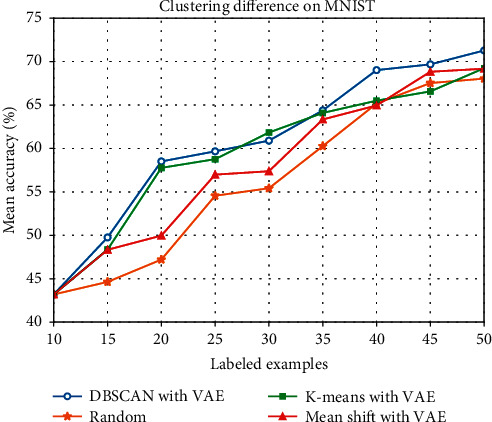
Mean test accuracy (%) of different clustering methods on MNIST.

**Figure 8:**
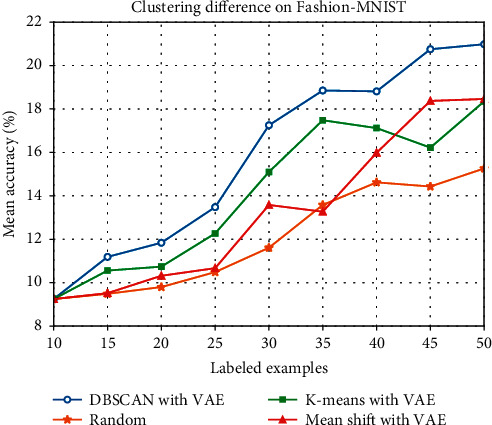
Mean test accuracy (%) of different clustering methods on Fashion-MNIST.

Furthermore, we replaced DBSCAN by K-means and Mean shift in order to study the different effects caused by different clustering methods. We also combined them with VAE and did experiment on MNIST and Fashion-MNIST. Detailed experimental results are listed in Tables 3 and 4, respectively. And we plot their performance in Figures 7 and 8.

However, what we may notice that, in Figure 7, the result of K-means with VAE is better than our method when the number of labeled data reaches 30 on MNIST. DBSCAN and K-means perform similarly until size 35 and DBSCAN performs clearly better after size 40. The reason may be that, in this quantity case, a great part of samples sampled by K-means method are closer to the potential characteristics of the labeled data than the data sampled by DBSCAN. In other side, this situation is probably caused by the uneven distribution of the samples when mapping the high-dimensional image data into the two-dimensional latent space. After size 40, DBSCAN may be more sensitive to detect the outlier points in latent space and prevent them being clustered. So the performance of DBSCAN is better than that of K-means when size is greater than 40. Of course, this is a hypothetical explanation. Probably, it is difficult to know for sure why K-means starts performing worse after size 40. However, on CIFAR-10 in Figure 8, compared with random sampling and the other two clustering methods, DBSCAN with VAE behaves better and the performance of increasing accuracy is more stable. It is somewhat clear from the results that our proposed method performs well on the whole on MNIST and Fashion-MNIST.

**Figure 9:**
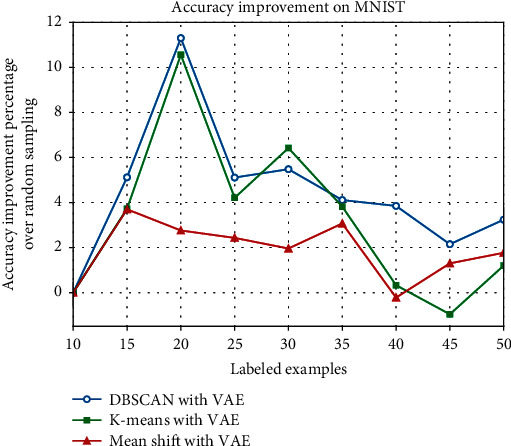
Accuracy improvement percentage over random sampling on MNIST.

**Figure 10:**
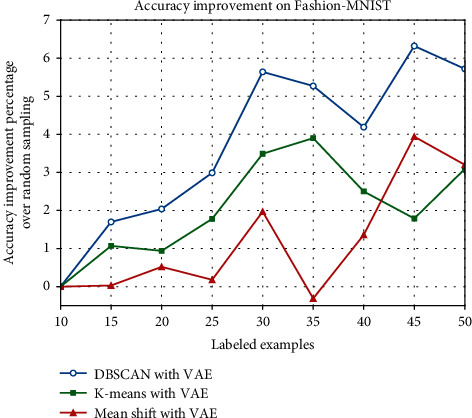
Accuracy improvement percentage over random sampling on Fashion-MNIST.

In order to show our experimental results more significantly, the improvement percentage of different clustering methods over random sampling is listed in Tables 5 and 6, and we plot their performance in Figures 9 and 10. On MNIST, our method DBSCAN with VAE, achieves the best results when the size of labeled samples reaches 20, increasing the accuracy by 11.30% over random sampling. Generally speaking, our approach performs even better in the other labeled data quantities. However, on Fashion-MNIST, our approach performs clearly better than the other two clustering methods at every labeled size in our experiment. And it achieves the best improvement of 6.32% when the labeled samples size reaches 45.

**Figure 11:**
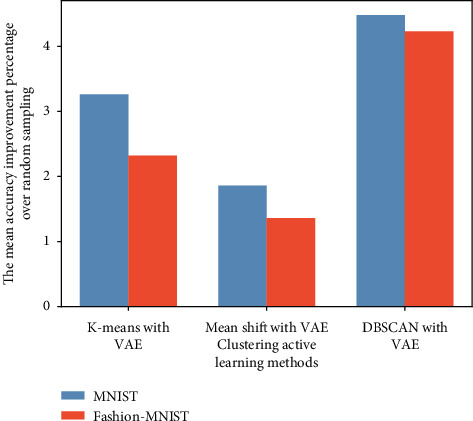
Mean accuracy improvement percentage on MNIST and Fashion-MNIST.

In the end, we computed the average accuracy improvement of each clustering method combined with VAE to see their overall improvement over random sampling, and we plot them in Figure 11. On MNIST, K-means and Mean shift achieve the improvement of 3.26% and 1.86%, respectively. And our method achieves the best performance of 4.48%. And on Fashion-MNIST, the three clustering active learning methods achieve the improvement over random sampling by 3.23%, 1.36%, and 4.23% in order. In a word, our proposed algorithm, DBSCAN with VAE, finally shows its superiority than K-means and Mean shift when combined with VAE on a relatively small query batch size on MNIST and Fashion-MNIST.

## 5. Conclusion and Discussion

In this paper, we proposed a new active learning method based on VAE and DBSCAN designed for neural networks with a relatively small query batch size. It overcomes the difficulty of distance representation in high dimensions and prevents the distance concentration phenomenon from occurring in the computational learning literature with respect to high-dimensional p-norms. Based on the results on MNIST, Fashion-MNIST, and CIFAR-10, we empirically show that our method achieves competitive performance compared with four common active learning methods listed above, and it is also superior to the other two clustering active learning methods for image classification when the query batch size is relatively small. For active learning models are usually based on a relatively large query batch sizes, our approach of small query batch sizes can be regarded as a supplement to the previous research studies. In addition, our method is simple to implement and can conceptually be extended to other domains, and we see it as a welcome addition to the arsenal of methods in use today. Furthermore, the inadequacy of our method is that manual parameter adjustment is required to make the total number of unlabeled points in all density-reachable clusters to be as close as possible to the needed amount of data for annotations in DBSCAN. And we will still thoroughly study the related work presented in this paper in the future.

## Figures and Tables

**Algorithm 1:**
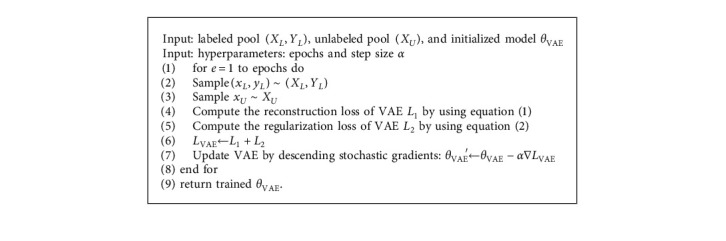
Variational autoencoder for active learning.

**Algorithm 2:**
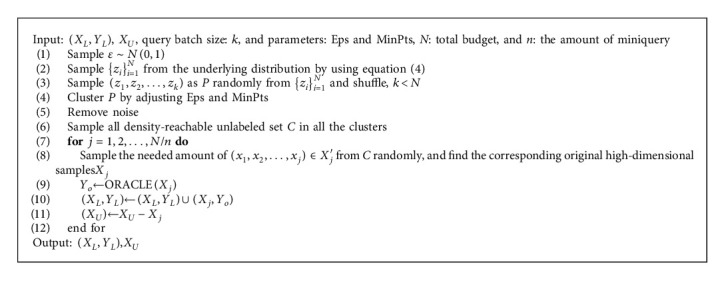
Sampling strategy by DBSCAN in the proposed model.

**Table 1:** Mean accuracy (%) of different methods on MNIST from 20 runs.

Number of labeled examples	15	20	25	30	35	40	45	50

DBSCAN with VAE	49.76	58.51	59.67	60.90	64.38	69.02	69.68	71.28
Random	30.19	43.05	52.34	51.68	57.30	64.03	60.75	64.96
Uncertainty	37.74	48.29	44.15	59.35	61.41	67.69	61.70	68.59
Core Set	49.03	48.03	54.51	54.99	56.34	58.89	53.97	60.13
EGL	44.73	46.80	41.27	52.37	49.89	51.49	57.05	59.73
Bayesian	32.33	31.50	35.30	37.59	39.52	45.13	54.41	56.21

**Table 2:** Mean accuracy (%) of different methods on CIFAR-10 from 20 runs.

Number of labeled examples	15	20	25	30	35	40	45	50

DBSCAN with VAE	11.19	11.84	13.48	17.25	18.85	18.81	20.75	20.98
Random	11.10	11.50	10.17	11.60	12.63	12.40	16.64	18.27
Uncertainty	10.22	11.87	12.87	12.92	13.91	13.95	15.56	20.24
Core Set	9.70	10.43	11.17	10.32	10.82	13.28	12.36	13.65
EGL	11.21	9.41	13.71	15.09	16.09	15.01	19.02	18.31
Bayesian	8.61	10.34	12.66	13.01	13.15	14.91	17.61	18.12

**Table 3:** Mean test accuracy (%) of different clustering methods on MNIST.

Number of labeled examples	Random (%)	K-means with VAE (%)	Mean shift with VAE (%)	DBSCAN with VAE (%)

15	44.6	48.36	48.34	49.76
20	47.2	57.77	49.97	58.51
25	54.56	58.77	56.99	59.67
30	55.42	61.68	57.38	60.90
35	60.27	64.09	63.34	64.38
40	65.17	65.50	64.95	69.02
45	67.53	66.57	68.83	69.68
50	68.04	69.24	69.18	71.28

**Table 4:** Mean test accuracy (%) of different clustering methods on Fashion-MNIST.

Number of labeled examples	Random (%)	K-means with VAE (%)	Mean shift with VAE (%)	DBSCAN with VAE (%)

15	9.49	10.56	9.52	11.19
20	9.80	10.74	10.32	11.84
25	10.49	12.27	10.67	13.48
30	11.61	15.10	13.58	17.25
35	13.58	17.48	13.27	18.85
40	14.62	17.12	15.98	18.81
45	14.43	16.22	18.37	20.75
50	15.26	18.35	18.46	20.98

**Table 5:** Accuracy improvement percentage over random sampling on MNIST.

Number of labeled examples	K-means (%)	Mean shift (%)	DBSCAN with VAE (%)

15	3.72	3.70	5.12
20	10.56	2.76	11.30
25	4.21	2.43	5.11
30	6.42	1.96	5.48
35	3.82	3.07	4.11
40	0.33	−0.22	3.85
45	−0.96	1.30	2.15
50	1.20	1.77	3.24

**Table 6:** Accuracy improvement percentage over random sampling on Fashion-MNIST.

Number of labeled examples	K-means (%)	Mean shift (%)	DBSCAN with VAE (%)

15	1.07	0.03	1.70
20	0.94	0.52	2.04
25	1.78	0.18	2.99
30	3.49	1.97	5.64
35	3.90	-0.31	5.27
40	2.50	1.36	4.19
45	1.79	3.94	6.32
50	3.09	3.20	5.72

## Data Availability

The data used to support the findings of this study are available from the corresponding author upon request.
